# Bisphosphonates Reduce Smoking-Induced Osteoporotic-Like Alterations by Regulating RANKL/OPG in an Osteoblast and Osteoclast Co-Culture Model

**DOI:** 10.3390/ijms22010053

**Published:** 2020-12-23

**Authors:** Sheng Zhu, Victor Häussling, Romina H. Aspera-Werz, Tao Chen, Bianca Braun, Weidong Weng, Tina Histing, Andreas K. Nussler

**Affiliations:** Department of Trauma and Reconstructive Surgery, BG Trauma Center Tuebingen, Siegfried Weller Institute for Trauma Research, Eberhard Karls University Tuebingen, 72076 Tuebingen, Germany; zhusheng8686@gmail.com (S.Z.); victor.haeussling@student.uni-tuebingen.de (V.H.); rominaaspera@hotmail.com (R.H.A.-W.); zzuchentao@yahoo.com (T.C.); bibi.braun@gmx.de (B.B.); wengweidong5657@gmail.com (W.W.); thisting@bgu-tuebingen.de (T.H.)

**Keywords:** co-culture, osteoblasts, osteoclasts, cigarette smoking, bisphosphonates, osteoporosis

## Abstract

Co-culture models have become mandatory for obtaining better insights into bone homeostasis, which relies on the balance between osteoblasts and osteoclasts. Cigarette smoking (CS) has been proven to increase the risk of osteoporosis; however, there is currently no proven treatment for osteoporosis in smokers excluding cessation. Bisphosphonates (BPs) are classical anti-osteoclastic drugs that are commonly used in examining the suitability of bone co-culture systems in vitro as well as to verify the response to osteoporotic stimuli. In the present study, we tested the effects of BPs on cigarette smoke extract (CSE)-affected cells in the co-culture of osteoblasts and osteoclasts. Our results showed that BPs were able to reduce CSE-induced osteoporotic alterations in the co-culture of osteoblasts and osteoclasts such as decreased matrix remodeling, enhanced osteoclast activation, and an up-regulated receptor activator of nuclear factor (NF)-kB-ligand (RANKL)/osteoprotegerin (OPG) ratio. In summary, BPs may be an effective alternative therapy for reversing osteoporotic alterations in smokers, and the potential mechanism is through modulation of the RANKL/OPG ratio.

## 1. Introduction

Bone tissue maintains its integrity by continuously regenerating itself tissue [1]. In general, approximately 10% of the mineralized bone is renewed every year. A balance between the bone-forming cells and the bone-resorbing cells is crucial to bone homeostasis. Osteoblasts as the bone-forming cells not only play a dominant role in bone formation but also regulate osteoclast differentiation through soluble factors and cognate interactions, which result in bone resorption [2]. The mechanisms regulating communication between osteoblasts and osteoclasts are demanding to the field of bone cell biology. Therefore, when trying to decipher the mechanisms underlying bone homeostasis, it is insufficient to study osteoblasts and osteoclasts separately. Co-culture models become mandatory in order to obtain better insight into the interactions between osteoblasts and osteoclasts [3]. However, certain technical challenges relating to co-culture models remain to be conquered, such as cell line compatibility, distinguishing between cell types, and selecting proper readouts [4]. It is of great value to optimize co-culture models of osteoblasts and osteoclasts to better understand the pathogenesis of bone diseases and explore potential treatments [5].

Cigarette smoking (CS) is commonly known as an indispensable risk factor for osteoporosis and osteoporotic fracture [6,7]. In our previous studies, cigarette smoke extract (CSE) has been proven to induce the risk of osteoporosis partially via disruption of transforming growth factor beta (TGF-β) signaling, increased oxidative stress, and, consequently, impaired osteogenesis of mesenchymal stem cells [8,9,10]. However, the specific mechanisms by which cigarettes affect mature osteoblasts and osteoclasts are still unclear. In theory, more osteoclast-mediated bone resorption than osteoblast-mediated bone formation leads to osteoporotic bone alterations [11]. The alterations may occur as direct or indirect effects of CS constituents on osteoblastic bone formation, and/or osteoclastic bone resorption, resulting in an imbalance between osteoblasts and osteoclasts [12]. It has been shown that patients with osteoporosis who smoke have more complex pathology compared to the general population of osteoporotic patients [13]. Thus, the comprehensive effects of CSE on the co-culture of bone cells need to be further clarified, and potential treatments for smoking-induced osteoporosis are specifically worth discovering. With regard to osteoporosis in smokers, current management strategies including smoking cessation, exercise, and dietary therapy are not complied by most patients [14]. Antioxidants, such as resveratrol, have been shown to potentially reduce the adverse effects of CS upon bone health, but they have not been used clinically [15]. Thus, there is wide demand for a feasible and flexible in vitro cell culture model that allows the user to stimulate osteoporotic-like alterations in smokers while screening potential therapies. Bisphosphonates (BPs), the most clinically used and effective anti-resorptive medication, are commonly examined in mono-culture and co-culture models in order to verify a response to stimuli [16]. Zoledronate and alendronate are among the most prescribed nitrogen-containing BPs in clinical practice, which have been proven to inhibit enzymes in mevalonic acid metabolism in osteoclasts to achieve anti-osteoporotic effects [17]. It is of great interest to explore whether BPs have a therapeutic effect for osteoporotic smokers.

In the present study, we first established an in vitro supplement-free co-culture system of osteoblasts and osteoclasts using human cell lines. Then, we evaluated the effects of BPs (zoledronate and alendronate) on CSE-affected cells using our co-culture model and analyzed the mechanisms involved.

## 2. Results

### 2.1. Co-Cultures of Osteoblasts and Osteoclasts Were More Stable than Mono-Cultures, and Co-Cultures Showed More Pronounced Effects from the Investigated Substances than Mono-Cultures Did

**Figure 1:**
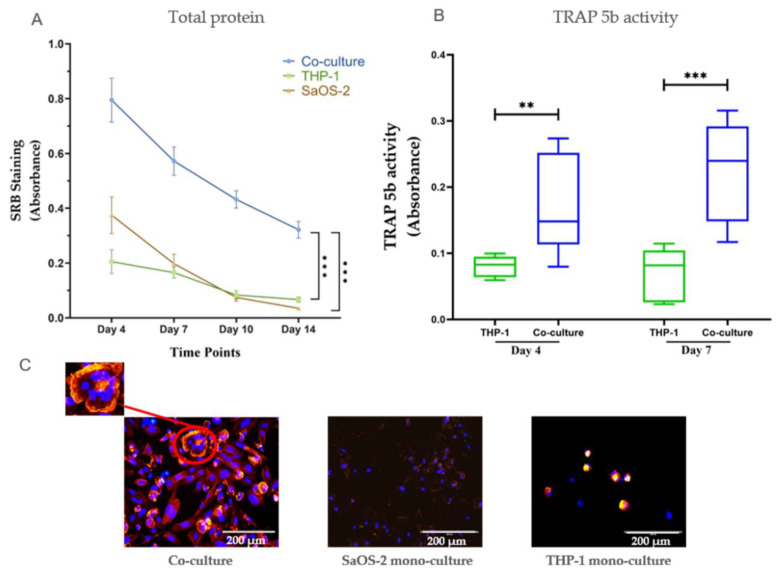
Comparison of SaOS-2 and THP-1 mono-culture and co-culture. (**A**) Sulforhodamine B (SRB) staining of co-cultures of SaOS-2 and THP-1 cells/THP-1 mono-culture/SaOS-2 mono-culture on day 4, 7, 10, and 14. (**B**) TRAP 5b activity of co-cultures of SaOS-2 and THP-1 cells and THP-1 cell mono-cultures. Data are represented the mean ± SEM, and the significance was determined as ** *p* < 0.01 and *** *p* < 0.001 (N = 3, n = 3). (**C**) Representative actin ring/nuclei staining in co-cultures/SaOS-2 mono-culture/THP-1 mono-culture on day 10. Osteoclasts were determined by the presence of actin ring formation and the presence of at least two nuclei, while osteoblasts were identified by actin filament structures and one nucleus.

We first compared the co-culture system with mono-cultures of human osteosarcoma cell line (SaOS-2) and human monocytic leukemia cell line (THP-1)cells. Cells in the co-culture model survived up to 14 days, while cell viability in mono-culture was significantly decreased after 7 days (Figure 1A). Tartrate-resistant acid phosphatase (TRAP) 5b activity, which is an important osteoclastic marker, was significantly higher in the co-cultures than that in the mono-culture (Figure 1B). According to fluorescence staining microscopy, we found multinucleated osteoclasts in the co-culture, proving that osteoclastic differentiation was successfully induced by osteoblast secretions in our co-culture system. Moreover, cells were unable to remain viable in mono-cultures after 10 days, whereas cell viability was maintained in co-cultures (Figure 1B).

### 2.2. CSE Had a Dose-Dependent Negative Effect on Cell Viability in Co-Cultures of Osteoblasts and Osteoclasts

**Figure 2:**
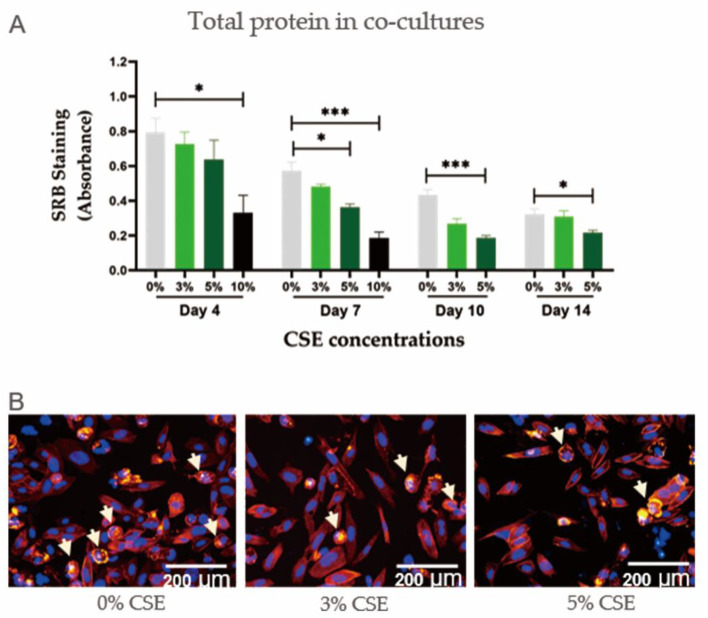
The effect of cigarette smoke extract (CSE) concentrations on cell viability in the co-culture of SaOS-2 and THP-1 cells. (**A**) SRB staining of co-cultures of SaOS-2 and THP-1 cells with exposure to CSE concentrations on day 4, 7, 10, and 14 (N ≥ 3, n = 3). Data are represented as the mean ± SEM, and the significance was represented as * *p* < 0.05, and *** *p* < 0.001 vs. 0% CSE group. (**B**) The representative actin ring/nuclei staining of co-cultures of SaOS-2 and THP-1 cells exposed to different CSE concentrations on day 10. The white arrows indicate actin rings which are formed by mature osteoclasts.

In order to evaluate the negative effects of CSE on our co-culture model, the system was exposed to CSE concentrations ranging from 0% to 10%. CSE showed a negative effect on co-culture cell viability in a dose-dependent manner (Figure 2A). CSE at a concentration of 10% was so toxic that the cell count fell by more than 50% on day 4 and 100% on day 7. CSE at a concentration of 5% also produced significant negative effects on co-cultures, although the cells did survive up to 14 days. Immunofluorescent staining confirmed the reduction in total cell numbers in co-cultures exposed to CSE (Figure 2B).

### 2.3. CSE Induced Osteoporotic-Like Alterations in Co-Cultures of Osteoblasts and Osteoclasts by Up-Regulating Osteoclastic Function

**Figure 3:**
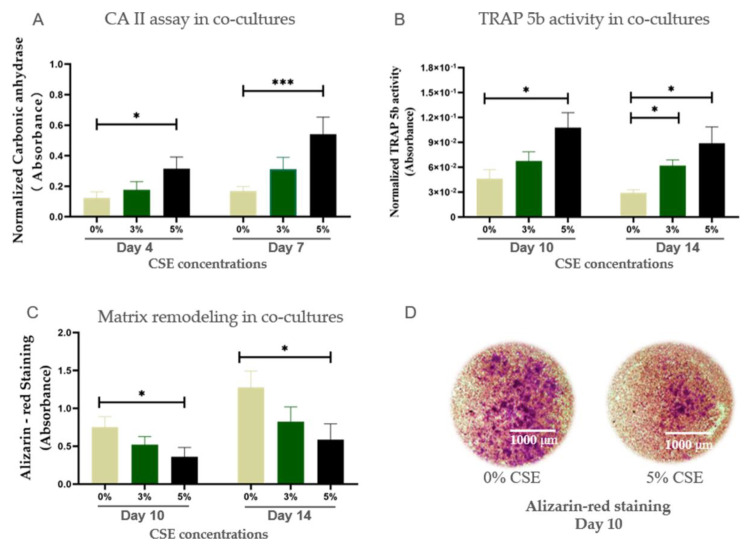
The effects of CSE concentrations on cell function and matrix mineralization in the co-culture of SaOS-2 and THP-1 cells. (**A**) CA II assay representing the osteoclastic differentiation of co-cultures. (**B**) TRAP 5b activity representing the mature osteoclastic function of co-culture of SaOS-2 and THP-1 cells on day 10 and day 14. (**C**) Alizarin red staining of co-culture of SaOS-2 and THP-1 cells on day 10 and day 14 (N ≥ 3, n = 3). (**D**) The representative microscopic representative images showed Alizarin red staining of 0% CSE (control group) and 5% CSE group on day 10 (N ≥ 3, n = 3). Data are representing the mean ± SEM. Significance was determinated as * *p* < 0.05, and *** *p* < 0.001 vs. 0% CSE group.

CA II is in characteristic for the early stage of osteoclastic differentiation and bone resorption [18]. Therefore, only results of CA II activity in the early time points (day 4 and day 7) are shown. TRAP 5b activity is utilized as a biochemical marker of osteoclast function [18] as well as a marker for the degree of bone resorption [19]. CSE at a concentration of 5% significantly up-regulated CA II and TRAP 5b activity in co-cultures (Figure 3A,B). Bone remodeling is associated with the resorption of mineralized bone by osteoclasts, followed by bone matrix formation by osteoblasts, which subsequently become mineralized [20]. The Alizarin red results showed that 5% CSE exposure had a significant negative effect on matrix remodeling in co-cultures (Figure 3C,D). Therefore 5% CSE was selected to use in the subsequent experiments.

### 2.4. BPs (Zoledronate and Alendronate) Counteracted the Effects of CSE on Co-Cultures of SaOS-2 and THP-1 Cells

**Figure 4:**
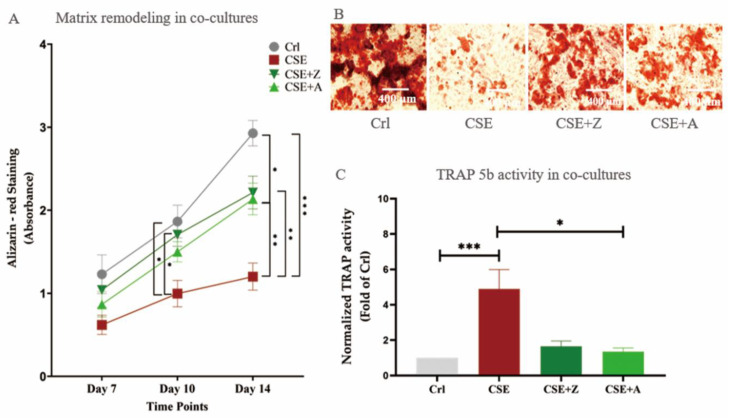
The effects of bisphosphonates (BPs) on CSE-affected cells in the co-culture of SaOS-2 and THP-1 cells. (**A**) Alizarin red staining of co-cultures exposed to 5% CSE with or *w*/*o* alendronate or zoledronate on day 7, 10, and 14. (**B**) A representative microscopy image showing Alizarin red staining of 0% CSE (Crl group) and 5% CSE group with or *w*/*o* alendronate or zoledronate on day 10. (**C**) TRAP 5b activity results of the co-cultures exposed to the same experimental set-up as for 7A and/B on day 7. Data are shown as the mean ± SEM, and the significance was set as * *p* < 0.05, ** *p* < 0.01 and *** *p* < 0.001 (N = 3, n = 3).

BPs are commonly used as anti-osteoporotic drugs, since they down-regulate osteoclast activity [21]. Due to the fact that increased osteoclastic activity is seen in CSE-exposed cells, we tested the possibility that BPs affected the negative outcomes regarding CSE-induced cellular damage in our co-culture setup [22]. As depicted in Figure 4, CSE (5%) significantly reduced bone matrix formation. In contrast, co-incubation with CSE and either alendronate or zoledronate resulted in significant improvements in matrix degradation compared to co-culture setups solely exposed to CSE. Additionally, zoledronate and alendronate reduced the TRAP 5b activity elevated by CSE, indicating that BPs could reverse the CSE-induced osteoclastic function on co-cultures.

### 2.5. CSE Exposure Enhanced Gene Expression of Osteoclastic Markers by Increasing the RANKL/OPG Raio, and BPs May Conteract the Effects of CSE on Co-Cultures

**Figure 5:**
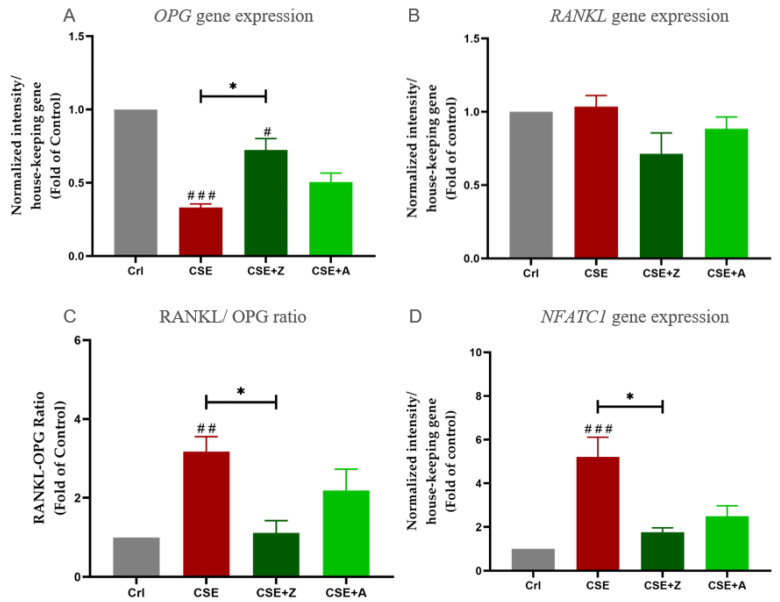
The effect of BPs on CSE-affected gene expression of osteoblastic and osteoclastic markers in the co-culture. (**A**) PCR of *OPG* gene expression in co-cultures exposed to CSE with or *w*/*o* zoledronate and alendronate on day 4. (**B**) PCR of *RANKL* gene expression in co-cultures exposed to CSE with or *w*/*o* zoledronate and alendronate on day 4. (**C**) The RANKL/OPG ratio of gene expression in co-cultures on day 4. (**D**) PCR of *NFATC1* gene expression in co-cultures exposed to CSE with or *w*/*o* zoledronate and alendronate on day 4. Data are shown as the mean ± SEM, and the significance was set as * *p* < 0.05, and # *p* <0.05, ## *p* <0.01, and ### *p* <0.001 vs. Crl group (N = 3, n ≥ 2).

The gene expressions of *OPG*, *RANKL*, and *NFATC1* were determined using PCR measurements. The receptor activator of nuclear factor (NF)-kB-ligand (RANKL)/osteoprotegerin (OPG) ratio is an important determinant of bone mass and skeletal integrity, which is pivotal in the regulation of osteoclast differentiation [23], and an elevated RANKL/OPG ratio suggests enhanced osteoclast activity [24]. In our experiments, β-actin was used as a house-keeping gene. CSE at a concentration of 5% down-regulated *OPG* gene expression; however, it increased *NFATC1* gene expression in the co-culture. Co-incubation with BPs counteracted the effects of CSE on *OPG* and *NFATC1* gene expression in co-cultures. Moreover, the RANKL/OPG ratio was significantly increased under CSE exposure, and co-incubation with zolendronate significantly reduced this up-regulation (Figure 5).

### 2.6. BPs Conteracted the Effects of CSE on Elevating Protein Expression of Osteoclastic Markers by Increasing the RANKL/OPG Ratio in Co-Cultures

**Figure 6:**
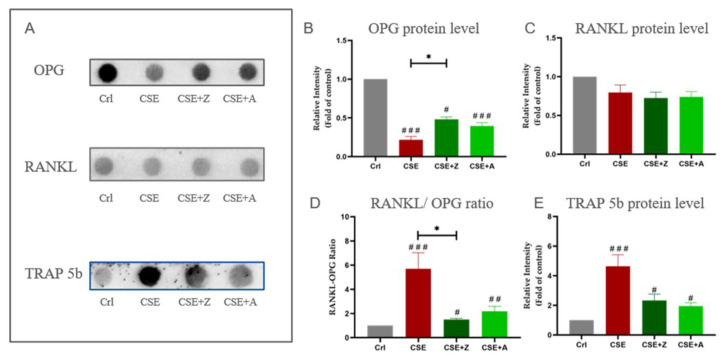
The effect of BPs on CSE-affected protein levels of osteoblastic and osteoclastic markers in the co-culture. (**A**) Representative dot blot images of protein levels of OPG, RANKL, and TRAP 5b. (**B**) Dot blot analysis of the OPG protein level in co-cultures exposed to CSE with or *w*/*o* zoledronate and alendronate on day 4. (**C**) Dot blot analysis of the RANKL protein level in co-cultures exposed to CSE with or *w*/*o* zoledronate and alendronate on day 4. (**D**) The RANKL/OPG ratio of protein levels in co-cultures on day 4. **E.** Dot blot analysis of the TRAP 5b protein level in co-cultures exposed to CSE with or *w*/*o* zoledronate and alendronate on day 4. Data are represented as the mean ± SEM, and the significance was represented as * *p* < 0.05, and # *p* <0.05, ## *p* <0.01, and ### *p* < 0.001 vs. Crl group (N = 3, n = 3).

Protein levels of RANKL, OPG, and TRAP 5b were measured using dot blot measurements. CSE at a concentration of 5% significantly decreased the OPG level; however, co-incubation with zoledronate and alendronate reduced this down-regulation. No significant difference of RANKL level was observed in all experimental groups. The RANKL/OPG ratio was calculated accordingly and showed that zoledronate and alendronate significantly reduced the CSE-induced up-regulation in the RANKL/OPG ratio. Moreover, CSE significantly increased TRAP 5b level, and both BPs were able to reduce this up-regulation caused by CSE (Figure 6).

## 3. Discussion

A tight balance between bone resorption and formation is required for healthy bone homeostasis. This balance is achieved not only by factors from the extracellular environment but also by comprehensive communications between osteoblasts and osteoclasts [25]. An imbalance in bone formation and resorption results in critical influences on bone mass and strength [26]. Therefore, it is mandatory to implement routine co-cultures consisting of osteoblasts and osteoclasts in order to gain better insight into the communications between cells as well as to screen or analyze potential treatment options for bone disorders [3,25]. Generally, co-culture models are characterized by the simultaneous cultivation of multiple cell populations, allowing for direct or indirect communication/contact between them [27]. Several studies have devoted to developing different co-culture systems of osteoblasts and osteoclats, but limitations still remain. On the one hand, indirect co-culture models of osteoblasts and osteoclasts such as Transwell device can not mimic the direct cell–cell contacts [28]. On the other hand, direct co-culture models of osteoblasts and osteclasts are able to mimic cell–cell contact; however, an effective method for distinguishing between the two cell types has not yet fully been resolved [29]. Moreover, osteoclastic differentiation induction require supplements such as RANKL and macrophage colony-stimulating factor (MCS-F) [30]. In the present study, we established a supplement-free co-culture model of osteoblasts and osteoclasts mimicking in vivo cell–cell contacts and applied a gender-specific DNA quantification method to distinguish different cell types. SaOS-2 cells were selected for the osteoblast precursor based on our previous study demonstrating advantages of maturity, matrix formation, and protein expression, while THP-1 cells were selected for the osteoclast precursor due to better compatibility and stability in a direct co-culture system [31]. In our study, THP-1 differentiation was directly induced by RANKL and MCS-F, which was secreted by SaOS-2 cells. Mature osteoblasts and multinucleated osteoclasts were observed in the co-cultures by fluorescent staining, demonstrating that the co-culture model is capable of achieving osteogenic and osteoclastic differentiation. The co-culture model had better cell survival and showed higher osteoclastic activity than the mono-cultures did. These results confirmed the priority of the co-culture model over mono-cultures and emphasized that communication between the two cell types plays a crucial role in cell survival and osteoclast differentiation.

CS is identified as an important risk factor for osteoporosis [32], and it has been shown to lead to a reduction in bone mass and the activation of osteoclastic markers in clinical studies [33,34]. In the present study, we evaluated CSE by using the co-culture model of osteoblasts and osteoclasts to verify its detrimental effects on bone health found in our in vivo study. In our study, CA II and TRAP 5b, which are considered biomarkers of functional osteoclasts [35], were up-regulated by CSE exposure in co-cultures. As a comprehensive result of the combined action of osteoblasts and osteoclasts, matrix remodeling was significantly decreased by CSE exposure in co-cultures. These CSE-induced changes in the co-culture are consistent with osteoporotic-like alterations in humans [36]. Moreover, consistent with the results described in in vivo studies [37,38,39], our results demonstrated dual actions of CSE on osteoblastic and osteoclastic markers at the gene and protein level. The down-regulated OPG expression and up-regulated osteoclastic markers induced by CSE are also in agreement with the pathogenesis of osteoporosis [40]. Therefore, CSE is considered a potent inducer of osteoporotic-like alterations in the co-culture of osteoblasts and osteoclasts and highlights that the co-culture model reflects the in vivo situation well.

BPs are commonly used to treat osteoporosis, and their effectiveness has been widely recognized [41]. Based on this, many in vitro experiments validate their cell culture system and bone cells function response using BPs [42,43]. The effects of BPs on CS-induced bone alterations have not been elucidated. Most studies reveal inhibitory effects of BPs on osteoclasts using mono-culture models, [44,45]. In our co-culture model, both tested BPs significantly mitigated the effects of CSE like decreased matrix remodeling and OPG expression, and enhanced osteoclastic function and expression of osteoclastic markers. Interestingly, BPs in our CSE-exposed co-culture model not only confirmed an inhibitory effect on osteoclasts but also showed a up-regulation of osteoblastic marker (OPG), which may be related to the interaction of the two types of cells in our co-culture system. These results suggest a clear role for BPs in reversing the osteoporotic alterations that is not limited to a single anti-osteoclastic effect. It has been shown that BPs affect osteoblasts by reducing matrix mineralization in a dose-dependent manner [46], while another study has demonstrated BPs to be promoters of osteoblast proliferation and maturation [47]. The anti-osteoporotic mechanisms by which BPs affect bone cells remain to be explored [48].

Bone remodeling is regulated by molecular interactions between RANKL and the decoy receptor OPG. The RANKL/OPG ratio is crucial for the regulation of osteoclast differentiation, activation, and survival, as well as the balance between bone formation and resorption [49]. Theoretically, a higher RANKL/OPG ratio suggests more differentiated and functional osteoclasts in vivo [50]. In the present co-culture study, we demonstrated that CSE had an inhibitory effect on OPG expression, leading to the significantly increased RANKL/OPG ratio. Our results are consistent with clinical data showing that long-term smokers have a significantly suppressed OPG production and an increased RANKL/OPG ratio [51]. The inhibition of osteoclastic differentiation via the mevalonate pathway is recognized as the main mechanism of BPs [52], but recent evidence indicates that BPs also regulate essential molecules related to osteoclastogenesis, such as the RANKL/RANK/OPG pathway [53,54]. However, the effects of BPs are still controversial regarding the RANKL/OPG ratio during bone remodeling. Some studies have found no obvious effects from BPs on RANKL/OPG [55,56]. One osteoblast mono-culture study has suggested that the RANKL/OPG expression is enhanced after stimulation by BPs [57], while a series of clinical studies have found that BPs may reduce RANKL expression and promote OPG expression in patients with osteoporosis [54,58]. In vitro cell culture models have also demonstrated that BPs enhance OPG expression and inhibit RANKL expression [59,60]. Moreover, another study has shown, for the first time, that BPs act at all three stages of bone remodeling, including modulating RANKL and OPG activity and, subsequently, osteoclastogenesis [61]. In our co-culture study, zoledronate and alendronate effectively increased OPG expression suppressed by CSE, resulting in significantly lower RANKL/OPG ratios compared to the CSE group. This could be a potential mechanism of the inhibitory effect of BPs on the osteoclastic differentiation and function elevated by CSE in the co-culture system. From this point of view, BPs could effectively be used for osteoporotic smokers or smoking-induced osteoporosis. Still, the effects of BPs on smoking patients need to be further validated in clinical trials, and particularly the adverse events associated with BPs, including hypocalcemia, musculoskeletal pain, osteonecrosis of the jaw, and atrial fibrillation would require close monitoring [62]. Furthermore, the details regarding the administration of BPs to smokers, including dosage, duration, and the necessity for intermittent administration need to be further investigated [63,64].

## 4. Materials and Methods

### 4.1. Cell Culture

#### 4.1.1. Culture of Cell Lines

SaOS-2 (DSMZ), which is an osteosarcoma cell line derived from the of an 11-year-old female, was used as a representative of osteogenic cells. THP-1 (DSMZ), which is a human leukemic cell line originated from a male patient with acute monocytic leukemia, was used as a representative of osteoclastic precursor cells. Both cells were cultivated in SaOS-2/THP-1 cell culture medium (RPMI 1640 Medium, 5% FCS). The medium was changed every 3–4 days. Sub-culture of SaOS-2 cells was performed at 80–90% confluence, and THP-1 cells were sub-cultured when the density reached 1 mio. cells/mL [65].

#### 4.1.2. Cell Seeding

Trypsin/Ethylenediaminetetraacetic acid (EDTA) was used to detach SaOS-2 cells. Viable SaoS-2 and THP-1 cells were stained with trypan blue and counted with a microscope. Cells were spun down by centrifuge (600× *g* for 10 min) and re-suspended with SaOS-2/THP-1 cell culture medium.

For the mono-culture of SaOS-2 cells, re-suspended cells were seeded in a 96-well plate (1 × 10^4^ cells per well). For the mono-culture of THP-1 cells, re-suspended cells were seeded with 200 nM phorbol-12-myristate 13-acetate (PMA) [8] in a 96-well plate (2 × 10^4^ cells per well).

For co-culture of SaOS-2 cells and THP-1 cells, re-suspended THP-1 cells containing 200 nM PMA were first seeded in a 96-well plate (2 × 10^4^ cells per well) to allow full adherence (37 °C, 5% CO_2_, humidified atmosphere). Re-suspended SaOS-2 cells were seeded in the same well (1 × 10^4^ cells per well) after THP-1 cells were attached and washed once with sterile PBS.

#### 4.1.3. Osteogenic Differentiation

For SaOS-2 cell differentiation, osteogenic medium (RPMI 1640, 2% fetal bovine serum (FCS), 5 mM β-glycerol phosphate, 200 μM L-ascorbic acid 2-phosphate, 1.5 mM CaCl_2_, 25 mM 4-(2-hydroxyethyl)-1-piperazineethanesulfonic acid (HEPES), and 5 μM cholecalciferol) was used to replace culture medium [66]. THP-1 cell differentiation was achieved by replacing culture medium by 50% fresh and 50% conditioned medium from well-differentiated SaOS-2 cells in a 6-well-plate (30 × 10^4^ cells per well) and cultured in parallel with THP-1 cells [67]. For co-cultures of SaOS-2 and THP-1 cells, the cell culture medium was replaced by SaOS-2 osteogenic medium, and secretions from differentiated SaOS-2 cells in the supernatant directly induced THP-1 cell differentiation.

### 4.2. Generation of CSE

One commercial cigarette (Marlboro, Philip Morris, New York, NY, USA) was continuously bubbled through a 25 mL pre-warmed RPMI 1640 medium in a standard gas wash bottle. Negative pressure generated by a peristaltic pump maintained the smoking process at a speed of 95–100 bubbles/min [68]. The CSE solution was determined photometrically (λ = 320 nm) by using a plate reader (BMG Labtech, Ortenberg, Germany). The CSE solution with an optical density of 0.7 was considered as 100% CSE. The CSE solution was sterilized by a 0.22 μm pore filter and diluted with SaOS-2 osteogenic medium to achieve different concentrations. In general, 0.1% CSE is associated with smoking slightly less than 0.01 pack cigarettes per day and 10% CSE stands for smoking 20 cigarettes per day [69].

### 4.3. Sulforhodamine B (SRB) Staining

SRB staining was used to determine total protein content. Cells were fixed with ethanol at −20 °C for at least 60 min. Ethanol-fixed cells were washed by PBS and incubated with 0.4% SRB solution (diluted in 1% acetic acid) under light protection for 30 min at room temperature. 1% acetic acid was used to remove unbound SRB. The bound SRB of cells was resolved with 10 mM trisaminomethane (TRIS) solution (pH = 10.5, Sigma-Aldrich, Darmstadt, Germany) and determined photometrically (λ = 565 nm) by using a plate reader after [9].

### 4.4. Actin and Nuclei Staining

Cells were washed once with PBS and fixed with 4% formaldehyde for 10 min. Cells were permeabilized with 0.2% Triton X-100 for 20 min and fixed with 2% formaldehyde for 10 min. After washing with PBS once, fixed cells were incubated with 5% BSA for 1 h to block nonspecific bindings. Osteoclast actin rings were visualized using Phalloidin-Tetramethylrhodamine (TRITC) (1:2000 in PBS) staining. The nuclei were stained by Hoechst 33,342 (1:1000 in PBS) staining, whereby blue fluorescence arises when intercalated into DNA. After staining, cells were washed with PBS. Osteoclasts were identified in the fluorescence microscope (Evos Fl, Thermo Fisher Scientific, Karlsruhe, Germany) by the presence of actin ring formation and at least two nuclei inside. Osteoblasts were identified by actin filament structures and one nucleus [70].

### 4.5. Carbonic Anhydrate II (CA II) Assay

Cells were washed with PBS and then incubated with CA II reaction buffer (12.5 mM TRIS pH = 7.5, 2 mM 4-nitrophenylacetate, and 75 mM sodium chloride). CA II activity was determined photometrically (λ = 405 nm) with a plate reader for 30 min continuously. Results were normalized to relative THP-1 cell number [71].

### 4.6. TRAP 5b Activity

For measuring TRAP 5b activity, 30 µL supernatant of cells was incubated with 90 µL TRAP 5b reaction buffer (pH = 5.5, 0.2% 4-nitrophenyl-phosphate, 100 mM sodium acetate, and 50 mM sodium tartrate) for 6 h at 37 °C. Then, 90 µL 1 M sodium hydroxide was used to stop the reaction. TRAP 5b activity was determined photometrically (λ = 405 nm) with a plate reader, and an osteogenic medium without cells was considered as the background control [72]. Results were normalized to relative THP-1 cell number.

### 4.7. Alizarin Red Staining

Matrix remodeling, a marker of the functional co-culture system, was measured by Alizarin red staining, which is commonly used to identify calcium mineralization and could reflect the comprehensive result from osteoblasts and osteoclasts function in the co-culture system [73]. Cells were first fixed with ethanol for at least 60 min at −20 °C. Cells were washed three time with tap water and incubated with Alizarin red solution (0.5% Alizarin Red S in ddH_2_O, pH = 4) for 30 min. Cells were washed with tap water three times and assessed microscopically. Cells were incubated with 100 µL 10% cetylpyridinium chloride for 20–30 min to resolve Alizarin red dye, and the quantification was determined photometrically (λ = 565 nm) with a plate reader after [74].

### 4.8. Gene Expression Analysis

Osteoprotegerin (OPG) and the receptor activator of nuclear factor (NF)-kB-ligand (RANKL), which are secreted by osteoblasts, regulate osteoclast formation and activation [75]. Nuclear factor of activated T cells 1 (NFATC1) is an important transcription factor secreted by osteoclasts that modulate osteoclastic differentiation and cell function [76].

PCR measurements were used to determine the gene expression of *OPG*, *RANKL,* and *NFATC1*. The total RNA of cells was isolated by Trifast reagent (0.4 mM ammonium thiocyanate, 0.8 mM guanidine thiocyanate, 3 M sodium acetate solution, and 0.68 mM glycerol). RNA concentration and purity check were determined by using a plate reader. Complementary DNA (cDNA) synthesis was performed using the cDNA synthesis kit from Thermo Fisher, and cDNA templates were diluted to 10 ng/µL in diethyl pyrocarbonate (DEPC) water. Red HS Taq Master Mix (Biozym, Vienna, Austria) was used for PCR reactions. In brief, a single 15 µL PCR reaction including 2 µL cDNA template, 4 µL DEPC water, 7.5 µL Red HS Taq Master Mix, and 0.75 µL forward and reverse primer (the information of used primers is shown in Table 1). The PCR was performed as previously described [66]. A 1.8% agarose gel mixed with ethidium bromide was used for samples loading (7.5 µL of each). Gel electrophoresis (85 V for 45 min) was carried out for the separation of the gels. PCR results were measured by the intensity of bands with ImageJ software (NIH, Bethesda, MD, USA). All the results of target genes were normalized to housekeeping gene (*β-Actin*) [74].

### 4.9. Protein Level Analysis

OPG, RANKL, and TRAP 5b are proteins that are secreted by osteoblasts or osteoclasts and presents in the supernatant of cells. Dot blot measurement was performed to determine protein levels into the supernatants. A dot blotter (Carl Roth, Karlsruhe, Germany) was used to apply supernatants (40 μL per well) of cells onto a wet nitrocellulose membrane. Ponceau staining was performed to confirm the transfer of proteins. 5% BSA in TBS-T (10% TRIS buffered saline (10x) TBS and 0.1% Tween-20 solution in ddH_2_O) was used to block the membranes. Membranes were washed with TBS-T and then incubated with primary antibodies against the target proteins at 4 °C for 24 h. After washing with TBS-T, the membranes were incubated with secondary antibodies (primary and secondary antibodies of target proteins are summarized in Table 2) for 2 h at room temperature. For signal development, the membranes were washed with TBS-T and then incubated with Enhanced Chemiluminescence (ECL) substrate solution. A Chemocam imager (INTAS, Göttingen, Germany) was used to detect the chemiluminescent signals, which were quantified by ImageJ software [65].

### 4.10. Total DNA Isolation and Quantification

Cells from the 96-well plate were washed once with PBS and incubated with 50 μL pre-warmed (up to 98 °C) 50 mM sodium hydroxide solution for 5 min. Supernatant was transferred to a reaction tube and incubated in thermoshaker for 30 min at 98 °C. 50 μL ddH_2_O and 5 μL 1M TRIS solution (pH = 8) was added into the reaction tube and centrifuged at 14,000× *g* for 10 min at 4 °C. The supernatant was transferred into a fresh Eppendorf safe-lock tube. For total DNA quantification, the same ratio of NaOH/ddH_2_O/1M TRIS was used as control. Then, 2 μL of the undiluted DNA sample was measured using the plate reader to obtain total DNA amount.

### 4.11. Cell-Type-Specific DNA Quantification for Co-Cultures

The THP-1 cell line derives from a male, while the SaOS-2 cell line is originally from a female. Based on that, gender-specific DNA quantification was used to normalize these two cell types in our co-culture model. As described, the THP-1 cell line derives from a male patient, while the SaOS-2 cell line originates from a female patient. The sex-determining region Y (SRY), which is the gene on the Y chromosome and only presents in male mammals [77], is used to determine the amount of THP-1 cells in the co-cultures. Samples from mono-culture of SaOS-2 cells were used as a negative control. The PCR measurement of SRY was performed to confirm gene expression changes for co-culture samples, and the PCR procedures were the same as mentioned above. A gradient number of mono-culture of THP-1 cells was used to make a standard curve by its DNA quantification and signal intensity of SRY. The relative DNA amount of THP-1 cells in co-cultures was calculated using the SRY expression and the standard curve [78]. The relative DNA amount of SaOS-2 cells in the co-culture was obtained by subtracting the relative DNA amount of THP-1 cells from the total DNA amount.

### 4.12. Statistics

Results are presented as mean ± SEM. GraphPad Prism Software 8.0 (El Camino Real, USA) was used for data analyses. Data were compared by non-parametric Kruskal–Wallis test followed by multiple comparison (Dunn’s test). *p* < 0.05 was considered as minimum level of significance. Biological (N) and technical (n) replicates are shown in the figure legends.

## 5. Conclusions

We established an in vitro supplement-free co-culture system with the direct contact of osteoblasts and osteoclasts using human cell lines that mimics the in vivo bone remodeling process. BPs (zoledronate and alendronate) were capable of significantly reducing the effects of CSE-induced osteoporotic-like alterations in the co-culture of osteoblasts and osteoclasts, suggesting that BPs may be an effective treatment for osteoporosis in smokers. In addition to the inhibitory effects on mature osteoclasts, a potential mechanism of BPs is to modulate the RANKL/OPG ratio elevated by CS.

## Figures and Tables

**Table 1:** Primer sequences and PCR conditions for the investigated genes.

Gene	Accession Number	Forward Primer (5′–3′)	Reverse Primer (5′–3′)	Product Length (bp)	Annealing Temperature (°C)	Cycles
*OPG*	NM_002546.3	CCGGAAACAGTGAATCAACTC	AGGTTAGCATGTCCAATGTG	313	60	35
*RANKL*	NM_033012.3	TCCCAAGTTCTCATACCCTGA	CATCCAGGAAATACATAACAC	245	56	35
*NFATC1*	NM_172390.2	TGCAAGCCGAATTCTCTGGT	CTTTACGGCGACGTCGTTTC	228	64	35
*β-Actin*	NM_001101.3	CGACAACGGTCCGGCATGT	GCACAGTGTGGGTGACCCCG	461	64	30

**Table 2:** Antibodies used in dot blot measurements.

Antibody	Catalog No.	Company	Dilution
OPG	500-P149	Peprotech	1:1000
RANKL	500-M46	Peprotech	1:1000
TRAP 5b	Sc-376875	Santa Cruz Biotech	1:1000
Goat anti-rabbit IgG-HRP	Sc-2004	Santa Cruz Biotech	1:10,000
Goat anti-mouse IgM	Sc-2064	Santa Cruz Biotech	1:10,000

## Abbreviations

BPs	Bisphosphonates
CA II	Carbonic anhydrate II
cDNA	Complementary DNA
CS	Cigarette smoking
CSE	Cigarette smoke extract
DEPC	Diethyl pyrocarbonate
ECL	Enhanced Chemiluminescence
EDTA	Ethylenediaminetetraacetic acid
MCS-F	Macrophage colony-stimulating factor
NFATC1	Nuclear factor of activated T cells 1
OPG	Osteoprotegerin
PMA	Phorbol-12-myristate 13-acetate
RANKL	Receptor activator of nuclear factor (NF)-kB-ligand
SaOS-2	Human osteosarcoma cell line
THP-1	Human monocytic leukemia cell line
TRAP	Tartrate-resistant acid phosphatase
TRITC	Tetramethylrhodamine
SRB	Sulforhodamine B
SRY	Sex-determining region Y
